# Youth engagement in mental health research: A systematic review

**DOI:** 10.1111/hex.13650

**Published:** 2022-11-16

**Authors:** Erin McCabe, Mungunzul (Megan) Amarbayan, Sarah Rabi, Justino Mendoza, Syeda Farwa Naqvi, Kalpana Thapa Bajgain, Jennifer D. Zwicker, Maria Santana

**Affiliations:** ^1^ Department of Pediatrics, School of Public Policy University of Calgary Calgary Alberta Canada; ^2^ Department of Medicine, School of Public Policy University of Calgary Calgary Alberta Canada; ^3^ Department of Community Health Sciences University of Calgary Calgary Alberta Canada; ^4^ Department of Biology, Faculty of Science and Technology Mount Royal University Calgary Alberta Canada; ^5^ Department of Social Policy and Health, School of Public Policy University of Calgary Calgary Alberta Canada; ^6^ Department of Pediatrics, Community Health Sciences University of Calgary Calgary Alberta Canada

**Keywords:** co‐design, community‐based research, mental health, mental health services, patient and public involvement, patient engagement, youth engagement

## Abstract

**Introduction:**

Patient engagement in youth mental health research has the potential to inform research on the interventions, services and policies that will benefit youth. At present, there is little evidence to guide mental health researchers on youth engagement. This systematic review aims to describe the impacts of youth engagement on mental health research and to summarize youth engagement in mental health research.

**Methods:**

We searched the following databases: MEDLINE, EMBASE and PsycINFO, using a combination of subject headings, keywords and synonyms for the concepts ‘patient engagement’, ‘youth’ and ‘mental health’. Articles that described engaging youth in mental health research were included. Two reviewers performed the study selection. Study characteristics, research activities performed by youth, impacts of youth engagement, challenges, and facilitators to engagement and recommendations for youth engagement described by authors were extracted. Quality appraisal involved determining the level of engagement of youth and the stage(s) of research where youth were involved.

**Results:**

The database search returned 2836 citations, 151 full‐text articles were screened and 16 articles, representing 14 studies, were selected for inclusion. Youth were involved at nearly all stages of the research cycle, in either advisory or co‐production roles. Youth engagement impacts included enhancing relevant research findings, data collection and analysis and dissemination to academic and stakeholder audiences. Both youth and academic researchers reported personal development across many domains. One negative impact reported was the increase in funding and resources needed for engagement. We produced a list of 35 recommendations under the headings of training, youth researcher composition, strategy, expectations, relationships, meeting approaches and engagement conditions.

**Conclusions:**

This study provides an understanding of the impacts and recommendations of youth engagement in mental health research. The findings from this study may encourage researchers to engage youth in their mental health research and support youth engagement in funding applications.

**Patient and Public Contribution:**

We consulted three youths with experience being engaged in mental health research about the review findings and the discussion. One youth designed a visual representation of the results and provided feedback on the manuscript. All youth's input informed the way the findings were presented and the focus of the discussion.

## 1 INTRODUCTION

Mental health conditions affect 1.2 million children and youth in Canada and this number is increasing.^1^ Five percent of Canadian children aged 5–17 years old report anxiety disorders and 2.1% reported a mood disorder in 2019.^2^ This aligns with the findings of a systematic review reporting on the prevalence of these disorders in high‐income countries (5.2% anxiety, 1.8% depressive disorder, 12.7% any mental health disorder).^3^ Of the 12.7% of children experiencing a mental health condition, only 44.2% received any services, revealing a large gap in services for children and youth mental health.^3^ Emergency department visits for paediatric mental health concerns have increased 61% from 2009 to 2019,^4^ which are often the result of a lack of availability of timely appointments in the community.^5^ It seems that current mental health services are not meeting the needs of children and youth, suggesting an urgent need to transform mental health services so that effective, accessible services are being provided.^3^, ^6^ As mental health services undergo a redesign, new innovative ways of implementing and delivering mental health care are being studied. It is important to involve youth in that research to ensure that practices, services, programmes and policies are appropriate, accessible and meet their needs.^7^ Using patient engagement in research is one approach to ensuring the youth perspective is integrated into mental health research and innovation.

The Canadian Institute for Health Research (CIHR) defines ‘patient engagement’ as the meaningful and active collaboration of individuals with personal experience of a health issue and their informal caregivers (including family and friends) in governance, priority setting, conducting research and knowledge translation activities.^8^ Patient engagement is a close equivalent of the United Kingdom's concept of Patient and Public Involvement.^9^ There is a growing acceptance of patient engagement as being essential in health research on the part of researchers, funders and research institutions. The arguments for patient engagement are philosophical (i.e., patients have a right to shape research about their condition), pragmatic (patient input improves the research process and relevancy of outputs) and practical (i.e., increased transparency and accountability for research that is produced by public funds).^10^

While patient engagement in adult health research is becoming well‐established, the momentum for youth patient engagement (herein, youth engagement) appears to be lagging (Mawn, 2015).^11^ This may be due to system‐level considerations for youth engagement, such as institutional research ethic board approval, issues of consent in youth and a lack of institutional support.^12^, ^13^, ^14^ It may also be due to practical issues such as researchers not feeling competent with youth‐friendly engagement methods, difficulties reaching youth for recruitment and funding issues.^12^ Also, the changing interests and developmental needs of youth may make it difficult to sustain engagement partnerships over the entire duration of a research project.^15^ Recruiting youth for mental health research may have additional challenges, as youth may have experienced stigma related to mental health in their community or within healthcare settings which may create issues of trust between youth and health researchers, leading to youth being reluctant to engage (Knaak, 2017).^16^ Youth may also be hesitant to disclose their mental health condition or may be concerned that their condition may become known to their peers as a consequence of their involvement in research. Furthermore, researchers may perceive youth with mental health conditions as vulnerable, and that research engagement activity may affect their well‐being.^14^

Despite these potential barriers, youth engagement is considered a guiding principle in recent efforts to redesign youth mental health services.^17^ Youth engagement allows researchers to gain important insights into why youth may not be accessing mental health services, create relevant and responsive interventions and create the conditions that make services accessible to young people.^18^ Youth engagement is also a way of recognizing youths' rights for agency and power in shaping mental health services that are for them.^19^ Learning about the benefits, successes, challenges and recommendations of researchers with experience with youth engagement in mental health research could help inspire researchers to engage youth in their own mental health research. Furthermore, an understanding of the impacts of youth engagement could support mental health funding applications where youth are engaged as research partners.

To date, the impacts of youth engagement on mental health research and the researchers have not been described. As well, while some recommendations exist about engaging youth in health research, there is little guidance for researchers about youth engagement specific to mental health research. Therefore, the primary purpose of this systematic review was to synthesize the impacts of youth engagement in mental health research. A secondary aim was to describe the challenges and facilitators encountered in mental health studies with youth engagement and to summarize the recommendations for youth engagement in mental health research made by authors.

## 2 METHODS

### 2.1 Study design

This systematic review follows the meta‐aggregative approach to qualitative synthesis outlined in the JBI Manual for Evidence Synthesis.^20^ JBI meta‐aggregative approach seeks to enable generalizable statements to guide practitioners and policymakers. It focuses on producing a synthesis of findings that authentically represent the aggregation of data from primary studies, rather than a more interpretive approach where authors re‐interpret findings from qualitative studies. The protocol for this review was registered with PROSPERO (CRD42022319240). We used the preferred reporting items for systematic review and meta‐analysis (PRISMA) guidelines to report this review.^21^ In this review, we distinguish youth co‐researchers from academic researchers by using the terms ‘youth researcher’ and ‘adult researcher’, respectively. We use the term ‘co‐production’ when referring to activities where youth are collaborating with adults or leading the activity, for example, developing recruitment materials. We use the term ‘advise’ to mean that youth researchers provided ideas and feedback on aspects of the project but were not directly involved in those activities. Three youths with experience engaging in youth mental health research were involved in this project.

### 2.2 Search

We searched MEDLINE, EMBASE and PsycINFO, using a combination of subject headings, keywords and synonyms for the concepts ‘patient engagement’, ‘youth’ and ‘mental health research’. The ‘patient engagement’ concept included participatory action research approaches, which are not always included in definitions of ‘patient engagement’, but were included here because they engage people who bring the collective voice of specific, affected communities to health research.^8^ We limited the search to 2000 to the present since patient engagement is a relatively new phenomenon in health research. The ‘mental health research’ concept included mental health, mental health services, as well as clinical diagnostic terms adapted from the Cochrane Common Mental Disorders Group with input from a pediatric psychiatrist. Duplicate citations were removed using automated software and manually by reviewers. Our search strategy is available online as Supporting Information: File 1.

### 2.3 Selection

#### 2.3.1 Inclusion and exclusion criteria

We included original research studies where youth were engaged as partners in the research process. We wanted to capture the variations in the approaches to including youth in mental health research, therefore we included a broad age range of youth researchers (8–25 years). To acknowledge that youth may be part of a research team over several years, we included articles where the majority of youth researchers were 25 years or younger. The age of the youth was assessed using the age at which the youth joined the team (where this information was available). Youth researchers could have lived experience with a mental health condition or not. All study contexts were included (i.e., mental health clinical research, mental health services research, community‐based participatory research or health promotion/public health research) and any setting (i.e., inpatient, outpatient, community, schools, residential treatment). We included studies conducted in countries with publicly funded health systems. The study must have described at minimum, one youth research activity and one impact of youth engagement.

We excluded articles that were not peer‐reviewed (e.g., commentaries, theses), those studying youth engagement in a programme of research (rather than a specific research project) and those where youth were engaged only in the stage of developing an intervention (e.g., mental health technology or clinical pathway) but not in research or evaluation of that intervention.

Two reviewers (E. M. and M. A.) screened citations on the title and abstract. The same reviewers reviewed the full text of the articles, comparing them against the inclusion criteria. At both stages, discrepancies between reviewers were resolved through discussion. Inter‐rater reliability was calculated using percent agreement and Cohen's *κ*. Covidence was used to manage the study selection process.

### 2.4 Quality appraisal

The focus of this review is on youth engagement within the research studies, and not the specific findings of each study. We felt that assessing the methodological quality of the studies themselves would be less meaningful than assessing the quality of engagement. However, to our knowledge, there are no quality assessment tools available to assess youth engagement as reported in a research article. Therefore, rather than an assessment of quality, we described youth engagement on two dimensions: level of youth engagement, and stages of the research cycle where youth were involved. The description of the level of youth engagement is based on the ‘Types of youth participation’ in *INNOVATE Research: Youth Engagement Guidebook for Researchers* (2019). These are *Participation* (i.e., youth are the subject of study), *Consultation* (i.e., youth provide feedback on research), *Partnership* (i.e., youth work collaboratively with researchers as equals) and *Youth‐led* (where every stage of research is driven by youth). Key stages in the research lifecycle are (1) Priority setting and planning; (2) Development of the research proposal; (3) Scientific review; (4) Ethics review; (5) Oversight of a research project; (5) Recruitment of research participants (for some types of research); (6) Data collection; (7) Data analysis and interpretation; (8) Knowledge exchange; (9) Evaluation and quality assurance.^22^ One reviewer (E. M.) categorized each study on these two dimensions, with a second reviewer verifying the descriptions (K. T. B.).

### 2.5 Data extraction and synthesis

Data extracted included study characteristics, characteristics of youth researchers, research activities of youth, as well as the findings of the study that related to youth engagement. We extracted findings about youth engagement for each of the following features: impacts of youth engagement on the research process and researchers, the facilitators and challenges to youth engagement and author recommendations for youth engagement. We used line‐by‐line extraction, from any location in the article, including methods, results, discussion and conclusions. Data extraction was performed by a single researcher (E. M.), with a second researcher cross‐checking the extracted data (K. T. B.). Discrepancies were resolved through discussion.

The findings for each feature were reviewed and descriptively coded. Codes were grouped by similarity in concept by a single reviewer and then combined into categories. One researcher (E. M.) created category descriptions, which were reviewed by one member of the research team (S. R.) and three youth researchers who were consulted.

### 2.6 Youth engagement in this review

We held a consulting meeting with three youths (ages 19–24, all identify as cis men, all Canadian citizens, one with Chinese and one with Southeast Asian heritage), all with previous experience engaging in mental health research. The aims of the consultation were threefold: to understand whether the way we presented the findings aligned with their experiences as youth engaged in research if they had additional recommendations for youth engagement and which of the findings were most salient to youth engaged in research. The feedback from the consultation informed how we presented the study's results and structured the discussion.

## 3 RESULTS

### 3.1 Search and selection

**Figure 1:**
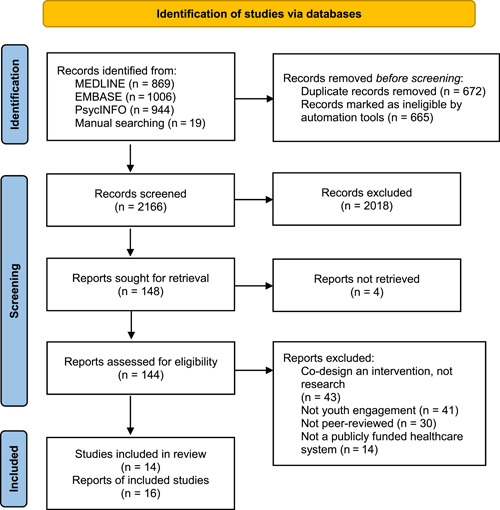
PRISMA diagram for article search and selection process

Figure 1 summarizes the search and selection process. The search retrieved 2838 citations. We removed 672 duplicates and 2166 citations were screened on the title and abstract. The percent agreement between authors was 88.4% (Cohen's *κ* = 0.52). The full‐text articles for 148 citations were reviewed, and 132 were excluded, primarily because they were describing co‐design of an intervention or clinical service (43 articles), or youth were participants in the study rather than involved as researchers (34 articles). Sixteen articles were included. The percent agreement between authors was 93.6% (Cohen's *κ* = 0.45). Two pairs of articles described the same study, therefore, a total of 14 studies were analysed.

### 3.2 Description of studies

Table 1 contains the key characteristics of the articles. The articles were published in four countries: Canada (*n* = 6), the United Kingdom (*n* = 8), Australia (*n* = 1) and Norway (*n* = 1). None of the articles were published before 2014 and most were published between 2020 and 2022 (*n* = 11). In nine articles, a description of youth engagement was embedded within the report on the research project, while seven articles reported directly on the youth engagement aspects of a research project.

**Table 1:** Characteristics of articles included in the analysis

Study ID	Year	Country	Sample characteristics	Sample age range	Sample size	Mental health research area	Study designs	Methods	Study setting
23	2020	United Kingdom	Children from a school in North West England	5‐10 years old	17 in focus groups, entire school for playtime observations	Public health research	Multiple methods	Focus groups and observation of play, participatory thematic analysis	School in North‐West England
24	2020	Canada	Varied in sex and gender, education, socioeconomic status, literacy, and mental health care experiences	16‐25	approximately 24	Health services research	Priority‐setting	Research priority setting	Community MH services, Hospital MH services
25	2020	Canada	Adolescents using child and adolescent mental health services close to transition and 1 year post transition	16‐19	21	Health services research	Qualitative study	Qualitative interviews	Child and adolescent mental health services in Ontario
26	2021	Canada	Youth who accessed CAMHS, caregivers of youth accessing CAMHS, and clinicians/administrators	youth 19‐25	20 youth, 17 caregivers, 21 clinicians and administrators	Health services research	Priority‐setting	Delphi study with qualitative analysis of comments	Child and adolescent mental health services in Ontario
27	2019	United Kingdom	Youth with severe mental illness, 81% female	18‐25 years old	16	Clinical research	Qualitative study	Qualitative interviews	Patients under the care of a community mental health team
28	2020	United Kingdom	Youth and adult researchers involved in a mental health research study with youth engagement	NR	NR	Clinical research	Descriptive study	Researcher reflections	Community mental health services
29	2021	Australia	Youth living in Youth Residential Rehabilitation Services	17‐25	18	Clinical research	Qualitative study	Qualitative interviews and focus groups	Youth Residential Rehabilitation Services
30	2017	United Kingdom	NA	NA	NA	Clinical research	Systematic review	Systematic review	Inpatient mental health services
31	2014	United Kingdom	Mixed ethnicity, genders, employment/education/unemployed	16‐25	65	Health services research	Participatory qualitative study	Quality standard development through focus group and nominal group technique	Community MH services, primary care, hostel, drop in service
32	2018	Canada	Youth accessing care for mental health concerns	NR	500	Health services research	Descriptive	Randomized controlled trial	Integrated community‐ based collaborative care team
33	2017	United Kingdom	2 male, 9 female, most in education	16‐18	11	Health services research	Multiple methods	Participatory design	Community
34	2021	Canada	Black youth living in Alberta, gender diverse, predominantly Christian	16‐30	30 interviews, 99 in conversation cafes	Public health research	Participatory qualitative study	Qualitative interviews and focus groups	Community
35	2021	United Kingdom	Students in 2 schools	18‐Nov	115	Public health research	Priority‐setting	Priority‐setting	Schools
36	2021	Canada	NR	NR	28	Health services research	Qualitative study	Qualitative study	Integrated community‐ based collaborative care team
37	2022	Norway	1 adult researcher and 10 youth researchers involved in youth engagement in research	adolescents over 15, adult	11	Health services research	Authoethnography	Autoethnography of patient engagement in a 4 year mental health research project	University of Stavanger
15	2021	United Kingdom	NA	NA	NA	Clinical research	Systematic review	Systematic review	NA

Study ID	Study primary purpose(s)	Key findings	Study focus	Number of youth researchers	Age ranges	Ethnicity	Gender/Sex	Youth with lived experience of mental health concerns	Model of youth engagement	Specific methods
23	How do playtime experiences impact social, emotional and mental health and well‐being, from the perspective of children?	Four themes: having someone to play with, games, things about playtime and how people treat each other.	Study report with engagement embedded	4	9‐10 years old	2 white, 1 chinese, 1 pakistani, all British born	2 boy 2 girls	No	Research and Development in Organisations (RADIO) model	Co‐produce
24	Aim to engage with youth and parents (or caregivers) in an emerging program of research aimed at understanding increasing mental health ED presentations in a Canadian paediatric tertiary health centre to support the development of effective interventions.	Research areas highlighted: (1) Access to mental health and addictions services; (2) Gaps in care; (3) Standards of care; (4) Stigma; (5) Experience of care; Also made recommendations for future engagement activities	Study report with engagement embedded	NR	NR	NR	NR	Yes	IAP2 Core Values for the Practice of Public Participation	Advisory meetings
25	To qualitatively explore the experiences of youth in relation to their knowledge, expectations, and experiences transitioning out of CAMHS services at age 18.	Themes: (1) Shifting awareness of the meaning of ‘transition’, (2) Ready or not to transition, (3) Mixed reactions to transitional age of 18 years, (4) Lack of information, preparation and involvement in the transition planning process, (5) Confusion around roles and responsibilities within the transition process, (6) Concern over transition gaps leading to poor mental health outcomes	Study report with engagement embedded	3	18+	NR	NR	Yes	McCain Model for Youth Engagement	NR
26	Prioritizing and refining the core components of effective transitions from child and adolescent to adult mental health services	26 core components; 3 themes: (1) need for youth and adult services to collaborate, (2) suggestions on how to operationalize core components, (3) barriers to implementation	Study report with engagement embedded	3	NR	NR	NR	Yes	NR	Advisory meetings and co‐produce
27	Explore the feasibility and acceptability of technologies to detect mental health deterioration	Four main themes: (1) dealing with mental health symptoms, (2) signs of mental health deterioration, (3) technology concerns and values and (4) technological applications to identify worsening mental health.	Study report with engagement embedded	7	18‐25 years old	5 White British, 1 British‐Asian, 1 Black‐British	2 male, 5 female,	Yes	NR	Advisory meetings and co‐produce
28	Explore the experiences and impacts of engagement in a qualitative study	Co‐producing research with youth makes a significant impact to the research, researchers and co‐researchers; co‐production takes time; build flexibility into budget and more interview training is recommended.	Study of engagement	7	18‐25 years old	5 White British, 1 British‐Asian, 1 Black‐British	2 male, 5 female,	Yes	NR	
29	Explore what matters to young people living in a 12‐month voluntary residential program for young people aged 16–25.	Two themes: factors that supported an environment for young people to thrive, and the ‘change work’ that young people undertook	Study report with engagement embedded	4	between 16‐25	NR	NR	Yes	Participatory action research informed by the critical emancipatory paradigm	Advisory meetings and co‐produce
30	Explore “risk” in inpatient mental healthcare with an evidence synthesis and input from stakeholders	Priority areas of “risk” of inpatient stays for MHC: Dislocation, Contagion, Harm from organisation, Institutionalisation, Self‐harm, Decision‐making, Suicide, Aggression, Other (in contrast to the clinical risks found in the literature ie self harm and aggression)	Study of engagement	2	under 18	NR	NR	Yes	Nominal group technique	Advisory meetings
31	To develop user‐generated quality standards for young people with mental health problems in primary care using a participatory research model.	16 quality standards for youth mental health in primary care	Study report with engagement embedded	29	16‐25	NR	NR	Yes	NR	Advisory meetings and co‐produce
32	For youth with mental health concerns, does an integrated collaborative care model, compared to usual care, result in better outcomes? Economic evaluation of the new model	A description of facilitators, barriers and youth researcher activities	Study of engagement	30	16‐26	NR	2 male, 6 female	Yes	McCain Model for Youth Engagement	Co‐biuld
33	To understand the emotional support related needs of young people.	Two ways of supporting young people's mental health: provide choice, raise awareness	Study of engagement	12	16	NR	2 male, 9 female	No	Nominal group technique	Advisory meetings
34	The purpose of this qualitative research study was to identify the barriers and facilitators to mental health care for Black youth in Alberta.	Barriers to mental health care access: lack of cultural safety and inclusion; lack of knowledge/information on mental health services; cost of mental health services and geographical and locational barriers; stigma and judgmentalism; and limits of resilience	Study report with engagement embedded	10	NR	Black	NR	No	Youth empowerment model situated within intersectionality theory	Meetings, co‐produce
35	Describes the establishment of a youth research advisory group to plan health research	Student health priorities centred on mental health and stress	Study report with engagement embedded	115	18‐Nov	“cultural and linguistic diversity”	100 female, 15 male	No	NR	Advisory meetings
36	Explore the research team's experience of youth and family engagement in the design of an RCT and clinical pathway.	A description of facilitators, barriers and recommendations for youth engagement in mental health research	Study of engagement	7	NR	NR	NR	Yes	McCain Model for Youth Engagement	Co‐produce
37	To understand the collaborative relationship between a lead researchers and youth researchers in a research project aiming to improving mental health services for adolescents.	6 themes: (1) Commitment motivated by altruism, personal interests and a common purpose, (2) Inclusiveness and support to reduce social uncertainty and strengthen collaboration, (3) Reduced power differentials while ensuring clarity of roles and task, (4) Diversity in representation to expand the perspectives of ‘the adolescent voice’, (5) Self‐determination ‐ supporting adolescents’ involvement in decision‐making processes, (6) Flexible and systematic project management.	Study of engagement	10	Adolescents over 15 years of age	“a variety of different ethnic backgrounds” and with different life and healthcare experiences	“both genders”	Mix	NR	Advisory meetings and co‐produce
15	Understand interventions for mental health in children with long term conditions.	Challenges, facilitators and recommendations for youth engagement	Study of engagement	8	17‐Oct	NR	3 male, 8 female	Yes	INVOLVE principles and values	Advisory meetings

The majority of studies engaged youth 16+ years old, with only one study engaging children 9–10 years old. Studies were on mental health services (*n* = 7), clinical research (*n* = 4) and public health (*n* = 3). Studies engaged between 2 and 115 youth. The studies with higher numbers of youth (*n* > 30) were priority‐setting and brainstorming‐type engagement activities. Five studies reported on the racial/ethnic diversity of the youth researchers, while seven reported on the sex or gender of engaged youth. A focus on diversity and inclusion within the research team was present in five studies. Most studies engaged youth with lived experience of mental health conditions (12/14). Five studies used advisory meetings as their only approach to engagement, while two studies engaged youth in specific research activities without conducting formal advisory meetings. Six studies used a combination of both advisory meetings and youth researchers engaging in specific research activities. A variety of models of youth engagement were used (see Table 1). Structured research training was provided to youth in five studies.

### 3.3 Youth engagement

The activities of youth researchers are described in Table 1. Youth were engaged as advisors and/or actively carried out specific research activities, in some cases leading the activities. Table 2 contains a summary of youth researcher activities, divided by whether the activity was done in a co‐production or advisory role. In four studies, the youth performed an advisory role only. The most common research activities were focusing on the research topic (*n* = 7), co‐analysis of qualitative data (*n* = 7) and dissemination of findings (*n* = 10).

**Table 2:** Research activities performed by youth researchers

Co‐production	Advisory role	References		
Co‐produce an agreement on roles and responsibilities for research team		[32, 36, 37]		
Co‐develop research design/protocol		[23, 27, 28, 32, 34, 36, 37]		
	Advise on scope of research, research design/focusing research question	[15, 23, 30, 34, 37]		
Co‐develop funding proposals		[30, 34, 37]		
	Advise on recruitment strategies	[26, 27, 28]		
Co‐develop study informational materials		[26, 27, 28, 32, 36, 37]		
Recruitment of participants		[34, 37]		
	Participate in advisory meeting(s)	[24, 30, 33, 35]		
Advise on environment/contextual factors for participant interactions	Advise on contextual factors and ways of relating for participant interactions	[24, 32, 36]		
	Advise on data collection instrument(s) (survey, interview guide)	[25, 26]		
Co‐develop data collection instrument(s) (survey, interview guide)		[27, 28, 29, 34, 37]		
Co‐facilitate focus groups/interviews/gather observational data from peers		[23, 27, 28, 29, 31, 34, 37]		
	Review content/thematic analysis and interpretation of findings	[15, 25, 26, 31]		
Co‐analysis of qualitative data		[23, 27, 28, 29, 31, 33, 34, 37]		
	Advise on dissemination strategies for stakeholders	[26]		
Present findings to stakeholders		[15, 23, 29, 34, 37]		
Co‐present at academic conferences		[15, 27, 28, 37]		
	Review journal manuscripts and final reports	[15, 33]		
Co‐write journal manuscripts and final reports		[25, 34, 37]		
Co‐produce recommendations for action based on research		[23, 31, 35]		

### 3.4 Quality appraisal

Youth were engaged at a ‘consultation’ level in five studies, a ‘partnership’ level in eight studies and one study was ‘youth‐led’. In three studies at the partnership level, a hybrid model was used where they had a small number of youth researchers were involved in research activities and a larger advisory committee of youth was consulted at key stages in the research process. This model was used to increase the diversity of the youth perspectives that influenced the research project. Table 3 contains the results of the quality appraisal, that is, the level of engagement of each study, and the stages of research where youth were involved. Seven studies involved youth in almost all stages of research.^23^, ^27^, ^28^, ^29^, ^32^, ^33^, ^34^, ^36^, ^37^ All studies involved youth in some form of quality assurance or evaluation of the research project, with five studies specifically involving youth in evaluating the engagement aspect of the project.

**Table 3:** A description of youth engagement by level of engagement and stage of research involvement

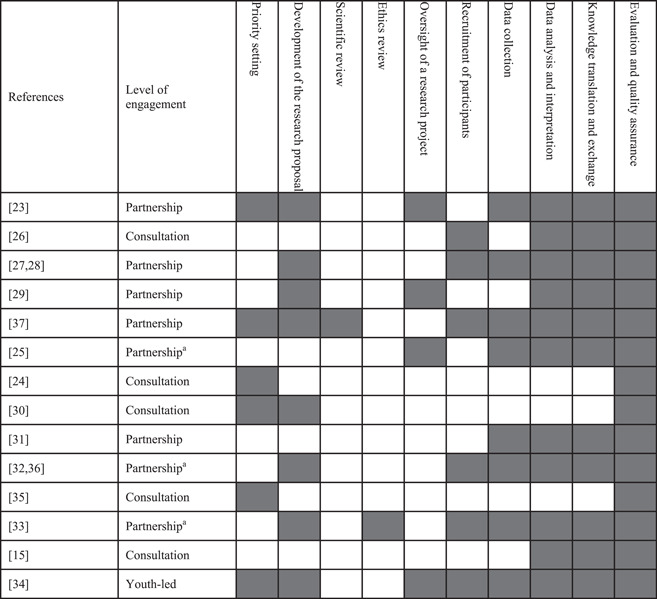

^a^Hybrid model of primary partnership with a small number of co‐researchers, with a larger advisory committee that was consulted for key stages in the research study.

### 3.5 Impacts of youth engagement

No studies reported a formal impact assessment of youth engagement, although four studies explored the impacts and experiences of youth engagement in research.^15^, ^28^, ^36^, ^37^ Table 4 contains a list of the impacts of youth engagement.

**Table 4:** Impacts of youth engagement on the research process and researchers

	Positive impacts	Negative impacts
Research process	Increase relevancy of research topics	More resources (time, effort, funding)
	Enhances safety and comfort for participants	
	Shape data collection and results^a^	
	Efficiency of decision‐making^a^	
	Enhance trustworthiness of findings	
	Enhanced dissemination of findings to academic, health system and patient audiences	
Personal impacts		
Youth researchers	Feeling empowered, respected, confident	
	Gaining knowledge about mental health and research	
	Social connectedness	
	Career development	
	Financial gain	
Youth and adult researchers	Expand network	
Adult researchers	An appreciation for youth engagement in research	Greater sense of responsibility
	Increased accountability for research	
	Sense of pride in youth researchers' development	

^a^ Reported as both positive and negative impacts in different articles.

The most common research process impacts of youth engagement reported by authors were (1) the data (*n* = 9), either by shaping the data collection instrument or being actively involved in data collection; (2) the findings from the study (*n* = 9), by youth involvement in the analysis; (3) enhanced knowledge dissemination (*n* = 9), by co‐presenting and advising on knowledge translation strategies. Enhancing the relevancy of research topics was another common impact reported in six studies, and four studies reported that having youth on the research team enhanced the safety and comfort of their research participants.^24^, ^27^, ^28^, ^32^, ^36^, ^37^ One study reported that youth engagement made decision‐making more efficient because youth provided perspectives that made the decision clearer.^32^, ^36^ Another study reported the opposite, that decision‐making was less efficient, but this was attributed to the adult research team members' intention to create an inclusive environment.^37^ Besides the efficiency of decision‐making, other negative impacts included the increased resources required for youth engagement (*n* = 6), and that youth may have unintentionally influenced data collection by asking leading questions or reassuring participants and sharing their own experiences.^27^, ^28^

Adult researchers reported increasing their knowledge of youth engagement strategies,^15^, ^27^, ^28^, ^32^, ^36^, ^37^ stating that youth engagement broadened their networks and enhanced their understanding of the research findings.^27^, ^28^ A sense of pride in the youth researchers' development over the course of the project was mentioned in two studies.^15^, ^37^ In one study, authors reported a greater sense of accountability for their research and thus more motivation to perform high‐quality research, which was described as positive.^15^ Related to this, in two studies, a greater sense of responsibility for youth researchers was reported as having a negative impact on adult researchers.^27^, ^28^, ^31^

**Figure 2:**
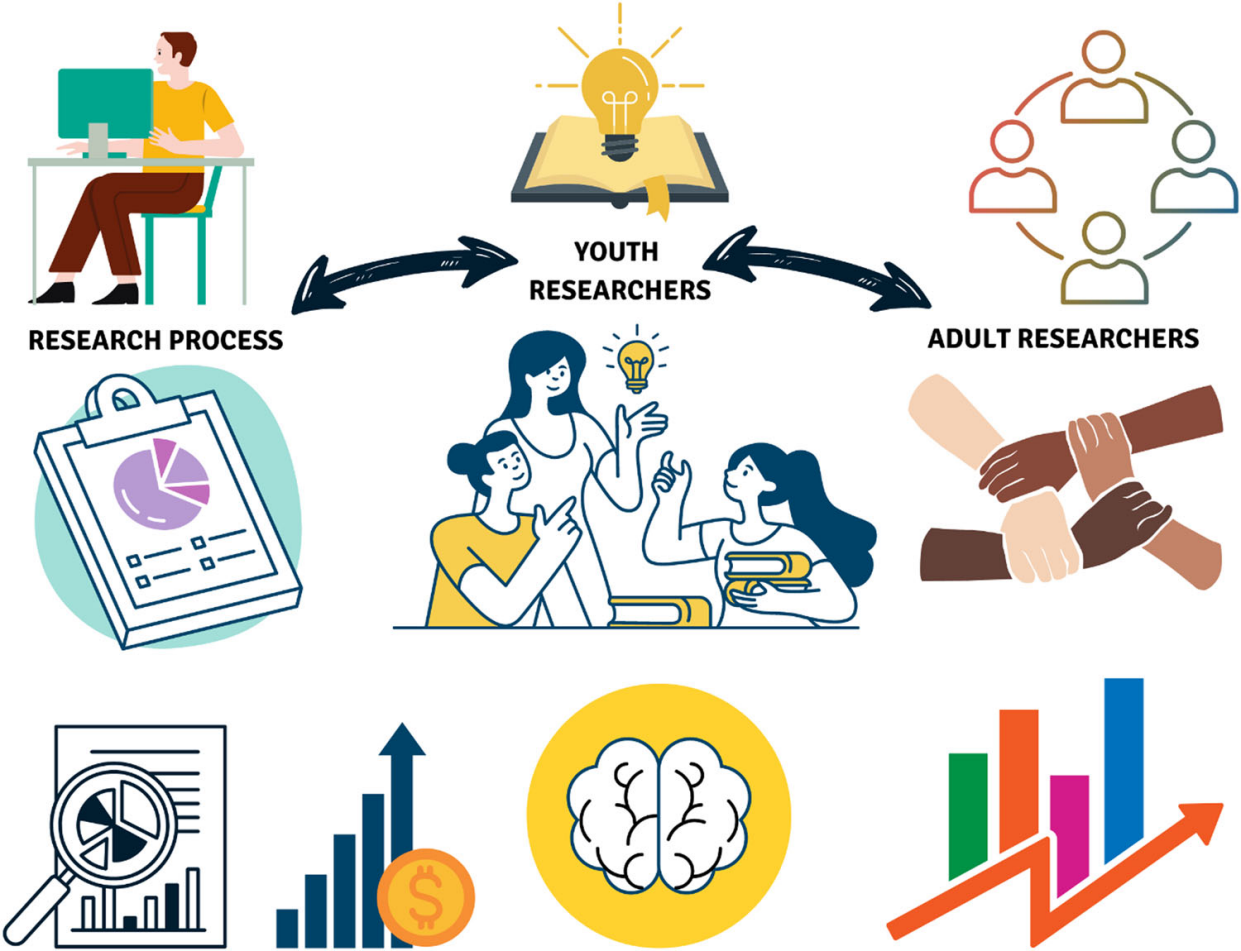
The impacts of youth engagement in mental health research. The two‐way arrows represent the effects that youth researchers have on the adult researchers and the research process and that also the adult researchers and being engaged in research has an impact on youth.

Youth researchers reported positive findings, feeling empowered and respected, particularly when witnessing their input being acted upon^15^, ^23^, ^29^ and increased confidence in their abilities.^27^, ^28^ They reported that they gained knowledge about research and mental health, and developed research, project management and communication skills.^15^, ^23^, ^27^, ^28^, ^37^ A sense of social connectedness and expanded networks were mentioned^15^, ^27^, ^28^, ^37^ as well as the research experience being a benefit for their job resumes and applications for postsecondary education and generating income.^37^ Figure 2 illustrates the impacts of youth engagement in research.

### 3.6 Facilitators and challenges to youth engagement

Table 5 describes the challenges and facilitators to meaningful youth engagement reported by the authors. One challenge reported in three studies was the time and effort for relationship‐building within the research team, and this was considered especially important in a mental health context.^15^, ^27^, ^28^, ^37^ There were challenges related to the recruitment and retention of youth researchers, and one study mentioned that as youth researchers become more skilled and acculturated to academic research environments, there was a need to monitor whether they were still representing the youth voice.^32^, ^36^ A final area of challenge related to navigating diverse perspectives and priorities of the research team. For example, adult researchers prioritize rigour versus youth wanting to reassure participants,^27^, ^28^ managing divergent youth and caregiver perspectives,^32^, ^36^ and perspectives of youth from different cultural backgrounds.^24^, ^32^, ^36^, ^37^

**Table 5:** A description of the facilitators and challenges to youth engagement

	Facilitators of youth engagement	Challenges of youth engagement
Relational	Create safe spaces Reflexivity in adult researchers (i.e., an awareness of power dynamics, how they are relating with youth) Efforts to build relationships (genuine, trusting) between youth and adult researchers Power‐sharing with youth (i.e., empowered in decision‐making, treating youth as equals) Using accessible language	More time/effort to build relationships, especially in mental health which can be a sensitive issue Power imbalance between youth and adults Communication barriers between adult and youth researchers Navigating diverse perspectives/conflicting priorities (adult vs. youth, youth vs. parents) Managing youth expectations (e.g., about the impact of the project)
Process	Using youth‐friendly communication tools (e.g., text messaging) Having a dedicated youth engagement coordinator Building relationships with community organizations Refreshments/ice‐breaking activities Flexibility with degree of involvement and scheduling Use of pre‐ and debriefs for large meetings Having diversity among youth voices Clear expectations for youth about engagement	More work to set up engagement (as a new process) More work to support (e.g., training, accommodating needs) and coordinate youth engagement More funding, time, work Recruitment of youth researchers (finding appropriate youth, representing diversity) Monitoring whether youth are remaining representative (as they become more involved in the project, youth researchers may begin to think more like adult researchers) Sustaining engagement over the course of the project Research ethics board Balancing bringing together a diversity of backgrounds and perspectives versus efficiency in decision making Not involving youth early enough to influence project Potential for youth engagement to affect research rigour

Relational facilitators of engagement included creating a safe, inclusive space for youth to share perspectives, adult researchers having an awareness of power dynamics and how they are relating with youth, and efforts to build genuine and trusting relationships. Process facilitators included having a dedicated youth engagement coordinator and providing refreshments and compensation for youth researchers.

### 3.7 Recommendations for youth engagement

Four articles contained recommendations for youth engagement in mental health research,^15^, ^27^, ^28^, ^36^, ^37^ while other articles contained recommendations embedded within Section 4. Table 6 contains a summary of recommendations for youth engagement. Recommendations were around training for both youth and adult researchers, the composition of the youth on the research team, processes for engagement, approaches to consultation meetings, agreement between youth and adult researchers about expectations, roles and responsibilities, elements of the relationship between youth and adult researchers and the conditions in which engagement occurs.

**Table 6:** Summary of recommendations for youth engagement in mental health research

Area	Recommendations
Training	Training should include education about the research topic, the research process and the opportunity to practice skills before project start. Training on communication and leadership skills should also be included.
	Training and support should be more intense early in the project, with a gradual reduction of support as youth competency increased.
	When transitioning youth into a project already in progress, be mindful that they are adequately prepared and have the same opportunity for training as youth who begin at the start of the project.
	Experienced youth researchers can lead youth research training.
	Enhance academic researchers' knowledge of youth engagement, for example, include patient engagement as part of a research Masters and PhD curriculum, provide additional training for established researchers.
Youth researcher composition	Consider recruiting several youth at the outset of the project due to difficulty sustaining youth involvement over time.
	Ensure diversity in youth representation when appropriate for the project, including diversity in research experience (include youth naïve to research).
Processes	Engage youth early in the research process to optimize their impact on the project.
	Have a dedicated engagement facilitator or share engagement coordination responsibilities with youth researchers.
	Be strategic about youth engagement activities, plan ahead for engagement during key transitions in research project when decisions will be made.
	Have a flexible budget with a contingency fund for unexpected research activities suggested by youth researchers.
	Build in a mechanism for asking for feedback from youth about the engagement process and how you will incorporate feedback into the process.
Meeting approaches	Provide opportunity for both written and verbal participation in the research process (e.g., nominal group technique, opportunities for written feedback if a youth cannot attend a meeting).
	Use age‐appropriate and engaging activities during consultation meetings.
	Consider having youth co‐facilitate meetings.
	When seeking feedback, use case scenarios and examples to make abstract concepts concrete.
	Use warm‐up activities before consultation meetings.
	Provide small group prebriefs for youth before meetings, explaining meeting objectives, key terms and an opportunity to ask questions.
	Hold small group debriefs after meetings, giving an opportunity to ask questions and provide feedback that youth were perhaps reluctant to share with a larger group of research team members.
	Provide refreshments.
Agreement on expectations	Be clear with youth about the objectives of the project and its expected impact.
	Establish clear role expectations, including the responsibilities of both the youth and adult researchers. This includes an agreement about the degree of control that youth have over the project.
Relational elements	To reduce power differential between youth and adults, establish a collaborative relationship between adult and youth researchers, on a foundation of trust, respect and rapport.
	Create a safe space for open discussion (e.g., include social identity in introductions, adult researchers being transparent and genuine).
	Dedicate time and funding for relationship building.
	Demonstrate respect for youth and their impact on the project by following through on their decisions and recommendations and sharing final results.
Engagement conditions	Consider ways of minimizing the potential for distress in youth (e.g., hold sessions at community agencies they are familiar with, provide peer and/or professional support, seek feedback from youth).
	Include caregivers but use separate forums to encourage youth's voice and unique opinions.
	Use youth‐friendly meeting spaces and communication tools (e.g., group messaging apps).
	Flexibility with meeting times and venues to accommodate youth schedules.
	Be flexible about the degree of involvement of youth.
	Be aware of and accommodate physical, mental and emotional needs of youth.
	Share power and leadership responsibilities with youth.
Incentives	Include incentives like course credits and certificates of completion where possible.
	Provide compensation for youth's time and travel for meeting and research activities.

### 3.8 Youth engagement in this review

Overall, the youth agreed with the findings of this review. They emphasized that overcoming the power differential between youth and adult researchers, as well as the representation of diverse youth voices was important. Their input resulted in the addition of one new impact, two new challenges, the reorganization of the recommendations section and the addition of concrete examples to some of the recommendations. We also revised the wording of some of the recommendations based on their feedback. One youth (J. M.) produced the visual of the impacts and also contributed to the writing of the manuscript, he is included as a co‐author on this paper.

## 4 DISCUSSION

Patient engagement research impacts have been conceptualized as both positive or negative, short or long‐term, and are either related to the research process (e.g., research instruments, outcomes measure choice, data collection design, delivery, time, dissemination) or impacts to the people involved (e.g., youth and adult researchers' experiences).^38^ Documented impacts of youth engagement on the research process include a positive influence on research design, recruitment, data collection and analysis and dissemination.^39^ It has also been reported to increase the youth friendliness and validity of research, the usability of practical tools, accessibility of consent forms and questionnaires and increase media attention.^7^, ^39^ There were few negative impacts reported, but inexperienced youth facilitators can negatively impact the quality of focus group data, and youth may interpret findings in relation to their own experiences impacting generalizability.^39^ Skill development, feeling empowered, confident and valued, as well as enhanced social connectedness, are positive impacts reported by youth engaged in research.^7^, ^39^ Academic researchers report an increased feeling of commitment to their project, inspiration and pride in their work.^39^ In this review, the impacts of youth engagement ranged from enhancing the relevancy of research topics to enhancing dissemination and impact on the health system. This aligns with what has been found in other reviews of youth engagement.^39^, ^40^ An impact unique to mental health research engagement was the enhanced comfort and emotional safety of research participants resulting from the involvement of youth. In one study, researchers used a pre‐engagement consultation with youth and caregivers to design a distress‐sensitive approach to their recruitment and data collection process, which included holding data collection sessions at community agencies with peer and professional support, providing written materials, giving participants the option of providing written feedback and to separate youth and caregivers.^24^ Another study reported that youth completing interviews were able to quickly develop rapport with participants and humanize the interview process for them. This was felt to enhance the emotional safety of participants, for whom talking about mental health may be uncomfortable or stressful.^28^

We found that youth researchers reported many personal benefits to being engaged in mental health research, including feeling empowered, a sense of social connectedness, gaining knowledge and skills and enhancing career and education opportunities.^15^, ^23^, ^28^, ^30^, ^37^ Youth researchers felt that research engagement expanded their professional networks, which was also reported by adult researchers.^28^, ^37^ The impact on adult researchers of engaging with youth was less often the focus of the studies, however, some impacts were reported such as gaining an appreciation for engagement, increased accountability for their research products and a sense of pride in youth researchers' development.^15^, ^28^, ^36^, ^37^ Adult researchers report that youth engagement added more to their responsibilities during research, because of their desire to foster positive engagement experiences for youth, which was viewed as both a positive and a negative impact.^15^, ^28^, ^31^

The negative impacts of youth engagement include the increased time and resources needed for engagement, which is commonly reported across all types of patient engagement studies.^39^, ^40^, ^41^, ^42^, ^43^ Researchers have reported concerns that youth with some mental health conditions could be vulnerable and engagement could potentially negatively impact their well‐being, whether from experiencing the power imbalance between adults and youths, or perhaps embedding the mental health condition as a part of a youth's identity.^14^, ^43^ We did not find evidence of these potentially negative impacts in our review, which may be reassuring for mental health researchers. Another potentially negative impact on the research relates to the methodological rigour of the research. Through their involvement in data collection and analysis, youth very commonly impacted data collection and analysis. This was viewed as positive in most cases, though there was some concern expressed about youth introducing bias into data collection and analysis through, for example, asking leading questions or incorporating their own experiences into data analysis.^28^ This was viewed by some as a negative impact, but one that could be overcome through training and close supervision.^28^ We also found that only one of the studies in this review used quantitative methods,^32^, ^36^ which could suggest that researchers believe quantitative studies are not suited to engagement or that youth engagement could limit the researchers' choice of methods to answer a particular research question. This was an issue that was also brought up by our youth researchers during the consultation meeting. However, outside of mental health research, youth have been engaged in quantitative research, for example, randomized controlled trials, comparative effectiveness research and measurement instrument development studies, which suggests that youth can be engaged in quantitative mental health research.^40^

There were practical challenges encountered by researchers engaging youth in mental health research. The increased resources that are needed for setting up and supporting engagement, recruiting and sustaining youth researchers throughout a project were mentioned across almost all studies Adult researchers also grappled with ethical considerations as well as navigating conflicting priorities of different groups, such as the youth and adult researchers, within youth researchers with different backgrounds and experiences, or between youth and caregivers.^24^, ^27^, ^28^, ^32^, ^36^ There were also challenges related to the relationship between the adult and youth researchers that needed to be overcome for productive working relationships to develop between youth and adult researchers. These included the inherent power imbalance between youth (as younger, novice researchers) and adults (as older, established researchers) and communication barriers between youth and adults. While these challenges are not unique to youth engagement in mental health research, authors felt that their importance was heightened in a mental health research context, which is a potentially sensitive subject.^15^, ^28^, ^36^, ^37^ Authors reported that putting in the time and effort to build trusting and genuine relationships was a successful way to overcome this challenge, as well as the adult researchers practising reflexivity (i.e., being self‐aware, reflecting on the way they relate to youth researchers). This finding aligns with the recent interest in the importance of relationships in patient engagement work.^44^, ^45^

The findings of this review support the idea that youth are willing and capable of being involved in research activities across the research cycle. Youth were involved, either in an advisory role or performing research activities, at all stages of CIHR's research cycle (i.e., from developing topics to disseminating findings). Studies reported successful youth engagement across all levels of engagement (Collaboration, Partnership, Youth‐led), which differs from some visions of patient engagement, where a partnership or complete control over research is considered the gold standard. This supports the idea put forth by Greenhalgh et al.^10^ that a more flexible approach to youth engagement, where the desired outcomes of engagement for the project and the motivations and capabilities of the individuals involved drive the engagement approach, rather than a single framework informing all patient engagement activities.

The recommendations contained in this article will be useful to researchers planning youth engagement in mental health research. They align well with the practical recommendations for youth engagement in health research put forth by Hawke et al.^7^ The recommendations from our review that might be considered unique to a mental health research context, such as creating a safe space for open discussion, accommodating emotional and mental needs, are incorporated in Hawke and colleagues' recommendations. The youth researchers we consulted in this review agreed with all the recommendations in the review. They emphasized the importance of overcoming power imbalances, which was a common theme among the articles in our review. They also felt that representation of diverse youth voices, in terms of ethnicity, race, gender and sexual identity and degree of experience in research was important. Related to this, they felt that adult researchers engaging with youth in a mental health context should have training in trauma‐informed approaches, as well as cultural competence. Although this was not a recommendation in any of the articles in this review study, it is supported by Shimmin and colleagues' argument that patient engagement should be underpinned by trauma‐informed approaches, as well as a recommendation in *INNOVATE Research*.^46^, ^47^ This may be especially true in a mental health context, where typically youth researchers are seeking to help shape a research project because of their experiential knowledge of mental health or mental health services. These experiences may co‐occur with traumatic experiences and asking the youth to share their experiences may be retraumatizing or cause them significant distress.^47^

### 4.1 Strengths, limitations and future directions

A strength of this review is the rigorous study search and selection strategy, and our focus on describing patient engagement in lieu of a traditional quality appraisal, which would have been less informative for this study. Also, we used an established method for aggregating qualitative findings.

A limitation of this review is the degree of youth engagement in the project. Youth were involved at the later stages of the review but were not involved in the conception or design of the review, which may limit the relevancy of this review for youth involved in research.

Also, as this is a relatively new field, the terminology used in the field of patient engagement varies across geographic settings. Though we made an effort to be comprehensive in our search strategy, there is the possibility that we missed some studies due to variability in terminology. As well, since this review relied upon authors' reporting on engagement activities, it is likely that some activities and impacts were missed, especially in studies where engagement was not the focus of the article.

One final limitation in this review is the possibility of a bias in our findings towards more positive engagement impacts. This could be due to adult researchers' position of power exerting control (intentionally or unintentionally) over what is reported in the manuscript leading to underreporting of negative experiences or impacts of youth engagement. Also, the inclusion criteria for this review included a requirement that authors reported on at least one activity and one impact on youth engagement. This may have created led to a positive bias in our findings because researchers who report more extensively about engagement may also have been more measured in their approaches to youth engagement, leading to positive engagement experiences for the research team. Similarly, due to the power imbalance between adult and youth researchers, youth researchers may be reluctant to report the negative impacts or experiences during the project. Finally, youth researchers could have experienced negative impacts in studies where youth engagement was minimally reported or where youth engagement was not evaluated. Therefore, our findings should be interpreted with some caution.

The impacts described in the articles were mostly proximal (e.g., effects of youth engagement on the research process), with some intermediate (e.g., skill development of researchers). However, the long‐term impacts of youth engagement, such as impacts on patient outcomes, were not reported. As previously discussed, none of the studies described a formal assessment of the impacts of youth engagement. This unfortunately limits the extent of the evidence for youth engagement in mental health research and also suggests a need for more formal evaluations of youth engagement in future projects. While impact assessment is complex and requires more resources, it is nevertheless important to lend credibility to the argument that patient engagement in research is worth the return on investment. To overcome the positive bias described above, these evaluations could be led by youth, giving them more power to openly report on engagement impacts.

## 5 CONCLUSION

The overall purpose of this systematic review was to synthesize the impacts of youth engagement on mental health research. We aggregated the reported impacts of youth engagement across research studies and described how youth were being engaged in research, challenges and facilitators to engagement. The recommendations for youth engagement in mental health research contained in this article can be applied by researchers who are planning to engage youth in mental health research. This study provides an understanding of youth engagement in mental health research that may encourage researchers to engage youth in their mental health research. It will also be useful in supporting requests for funding for youth engagement.

## AUTHOR CONTRIBUTIONS

Erin McCabe conceptualized and designed the study, search and selected articles performed data extraction and analysis and wrote most of the manuscript. Mungunzul (Megan) Amarbayan contributed to the study design, and article selection, and critically reviewed the manuscript. Sarah Rabi assisted with youth researcher consultations, and wrote parts of and critically reviewed the manuscript. Justino Mendoza contributed to the analysis and interpretation of results, developed a figure and critically reviewed the manuscript. Syeda Farwa Naqvi assisted with youth researcher consultations and critically reviewed the manuscript. Kalpana Thapa Bajgain performed data extraction and critically reviewed the manuscript. Jennifer D. Zwicker contributed to the study design, and data interpretation and critically reviewed the manuscript. Maria Santana conceptualized the study, contributed to study design and data interpretation and critically reviewed the manuscript. All authors reviewed and approved the final manuscript.

## CONFLICT OF INTEREST

The authors declare no conflict of interest.

## Supporting information

Supplementary information.

## Data Availability

Data are available on request from the corresponding author.
