# Supplementary material for: Youth engagement in mental health research: A systematic review
Source: Health Expect. 2022 Nov 16;26(1):30–50. doi: 10.1111/hex.13650 (PMC9854331; doi:10.1111/hex.13650)
Supplement: Supplementary file 1 — Supplementary information. [file HEX-26--s001.docx]

**Final Search Strategy for PE in CYMH Research**

Databases searched: MEDLINE, EMBASE, PsychINFO

Search concepts:

Child and youth

Patient engagement – terms from Flynn (2019) and Domecq (2014)

Mental health research terms – adapted from Cochrane Common Mental Disorders Group (<https://cmd.cochrane.org/search-strategies-identification-studies>) with input from paediatric psychiatrist (added psychosis, removed some terms like combat disorders, post partum)

Strategy built in consultation with a health sciences librarian

Time period: 2000 to present

**MEDLINE SEARCH**

Date: March 15, 2022

Total retrieved: 869 citations

Ovid MEDLINE(R) and Epub Ahead of Print, In-Process, In-Data-Review & Other Non-Indexed Citations and Daily <1946 to March 14, 2022>

| 1 | (Child* or pediatric* or paediatric* or teen* or adolesc* or youth* or young adult* or young people or young person).kf,tw. | 2043720 |
| --- | --- | --- |
| 2 | (advisory board* or advisory committee* or advisory group* or SPOR or strategy for patient-oriented research or patient-centred outcomes research institute or co-design or youth-adult partnership*).kf,tw. | 12505 |
| 3 | ((Patient* or famil* or parent* or caregiver* or stakeholder* or community or youth* or child* or adolesc* or public or consumer) adj2 (involv* or oriented or orientated or collaborat* or partner* or collaborat* or engage* or activat*) adj4 (research or trial or method* or health technology assessment or study)).kf,tw. | 20707 |
| 4 | Community-Based Participatory Research/ | 5231 |
| 5 | patient participation/ | 28327 |
| 6 | ((mental or psychiatric or psychologic*) adj (health or illness* or disorder* or disease* or problem or problems or issue* or concern* or well-being)).kf,tw. | 335561 |
| 7 | Mental Health Services/ | 36876 |
| 8 | Mental Health/ | 51173 |
| 9 | anxiety disorders/ or agoraphobia/ or anxiety, separation/ or neurotic disorders/ or obsessive-compulsive disorder/ or panic disorder/ or phobic disorders/ | 83426 |
| 10 | mood disorders/ or affective disorders, psychotic/ or bipolar disorder/ or depressive disorder/ or depressive disorder, major/ or depressive disorder, treatment resistant/ or dysthymic disorder/ or premenstrual dysphoric disorder/ or dysthymic disorder/ or seasonal affective disorder/ | 158338 |
| 11 | "trauma and stressor related disorders"/ or adjustment disorders/ or stress disorders, traumatic/ or psychological trauma/ or stress disorders, post traumatic/ or stress disorders, traumatic, acute/ | 44016 |
| 12 | mental disorders/ or impulse control disorders/ or personality disorders/ or somatoform disorders/ or body dysmorphic disorders/ or conversion disorder/ or hypochondriasis/ or neurasthenia/ | 207645 |
| 13 | Psychotic Disorders/ | 49774 |
| 14 | "feeding and eating disorders"/ or anorexia nervosa/ or avoidant restrictive food intake disorder/ or binge-eating disorder/ or bulimia nervosa/ or diabulimia/ or "feeding and eating disorders of childhood"/ or orthorexia nervosa/ or relative energy deficiency in sport/ | 32256 |
| 15 | self-injurious behavior/ or exp suicide/ | 75629 |
| 16 | (eating disorder* or anorexia nervosa or bulimi* or binge eat* or (self adj (injur* or mutilat*)) or suicide* or suicidal or parasuicid* or mood disorder* or affective disorder* or bipolar i or bipolar ii or (bipolar and (affective or disorder*)) or mania or manic or cyclothymic* or depression or depressive or dysthymi* or neurotic or neurosis or adjustment disorder* or antidepress* or anxiety disorder* or agoraphobia or obsess* or compulsi* or panic or phobi* or ptsd or posttrauma* or post trauma* or combat or somatoform or somati#ation or medical* unexplained or body dysmorphi* or conversion disorder or hypochondria* or neurastheni* or trichotillomania or affective symptoms or mental disorder* or mental health or psychosis or psychotic).ti. | 450475 |
| 17 | 2 or 3 or 4 or 5 | 64456 |
| 18 | 6 or 7 or 8 or 9 or 10 or 11 or 12 or 13 or 14 or 15 or 16 | 912760 |
| 19 | 1 and 17 and 18 | 934 |
| 20 | 19 | 934 |
| 21 | limit 20 to yr="2000 -Current" | 869 |

**EMBASE search**

Date: March 15, 2022

Total retrieved: 1006

Embase <1974 to 2022 March 14>

| 1 | (Child* or pediatric* or paediatric* or teen* or adolesc* or youth* or young adult* or young people or young person).kf,tw. | 2558220 |
| --- | --- | --- |
| 2 | (advisory board* or advisory committee* or advisory group* or SPOR or strategy for patient-oriented research or patient-centred outcomes research institute or co-design or youth-adult partnership*).kf,tw. | 23123 |
| 3 | ((Patient* or famil* or parent* or caregiver* or stakeholder* or community or youth* or child* or adolesc* or public or consumer) adj2 (involv* or oriented or orientated or collaborat* or partner* or collaborat* or engage* or activat*) adj4 (research or trial or method* or health technology assessment or study)).kf,tw. | 31131 |
| 4 | participatory action research/ or participatory research/ | 6731 |
| 5 | patient participation/ | 31495 |
| 6 | ((mental or psychiatric or psychologic*) adj (health or illness* or disorder* or disease* or problem or problems or issue* or concern* or well-being)).kf,tw. | 416205 |
| 7 | mental health care/ or mental health service/ or psychosocial care/ or mental health center/ | 108851 |
| 8 | mental health/ or community mental health/ or psychological well-being/ | 192637 |
| 9 | anxiety disorder/ or acute stress disorder/ or anxiety neurosis/ or cardiac anxiety/ or distress syndrome/ or generalized anxiety disorder/ or "mixed anxiety and depression"/ or panic/ or posttraumatic stress disorder/ or psychasthenia/ or separation anxiety/ or adjustment disorder/ or obsessive compulsive disorder/ or phobia/ or agoraphobia/ or claustrophobia/ or social phobia/ | 259804 |
| 10 | depression/ or agitated depression/ or atypical depression/ or depressive psychosis/ or dysphoria/ or dysthymia/ or endogenous depression/ or involutional depression/ or major depression/ or masked depression/ or melancholia/ or "mixed anxiety and depression"/ or "mixed depression and dementia"/ or mourning syndrome/ or organic depression/ or premenstrual dysphoric disorder/ or pseudodementia/ or reactive depression/ or recurrent brief depression/ or seasonal affective disorder/ or mood disorder/ or affective neurosis/ or blunted affect/ or depression/ or major affective disorder/ or mania/ or minor affective disorder/ or bipolar disorder/ or bipolar depression/ or bipolar i disorder/ or bipolar ii disorder/ or bipolar mania/ or cyclothymia/ or manic depressive psychosis/ or "mixed mania and depression"/ or rapid cycling bipolar disorder/ or mania/ or hypomania/ or manic psychosis/ | 571218 |
| 11 | mental stress/ | 91407 |
| 12 | emotional disorder/ or impulse control disorder/ or intermittent explosive disorder/ or kleptomania/ or pyromania/ or trichotillomania/ or somatoform disorder/ or body dysmorphic disorder/ or cardiac anxiety/ or conversion disorder/ or hypochondriasis/ or masked depression/ or psychogenic pain/ or somatic delusion/ or somatization/ | 49520 |
| 13 | psychosis/ or acute psychosis/ or affective psychosis/ or brief psychotic disorder/ or childhood psychosis/ or delusion/ or depressive psychosis/ or endogenous psychosis/ or hallucination/ or manic psychosis/ or paranoid psychosis/ or schizophrenia/ | 274544 |
| 14 | eating disorder/ or anorexia nervosa/ or binge-eating disorder/ or bulimia/ or orthorexia nervosa/ or female athlete triad/ | 53575 |
| 15 | automutilation/ or suicidal behaviour/ or self poisoning/ or suicidal ideation/ or suicide/ or suicide attempt/ | 126856 |
| 16 | (eating disorder* or anorexia nervosa or bulimi* or binge eat* or (self adj (injur* or mutilat*)) or suicide* or suicidal or parasuicid* or mood disorder* or affective disorder* or bipolar i or bipolar ii or (bipolar and (affective or disorder*)) or mania or manic or cyclothymic* or depression or depressive or dysthymi* or neurotic or neurosis or adjustment disorder* or antidepress* or anxiety disorder* or agoraphobia or obsess* or compulsi* or panic or phobi* or ptsd or posttrauma* or post trauma* or combat or somatoform or somati#ation or medical* unexplained or body dysmorphi* or conversion disorder or hypochondria* or neurastheni* or trichotillomania or affective symptoms or mental disorder* or mental health or psychosis or psychotic).ti. | 543577 |
| 17 | 2 or 3 or 4 or 5 | 89473 |
| 18 | 6 or 7 or 8 or 9 or 10 or 11 or 12 or 13 or 14 or 15 or 16 | 1509915 |
| 19 | 1 and 17 and 18 | 1838 |
| 20 | 19 | 1838 |
| 21 | limit 20 to yr="2000 -Current" | 1782 |
| 22 | limit 21 to embase | 1006 |

**PsycINFO Search**

Date: March 15, 2022

Total retrieved: 944

APA PsycInfo <1806 to March Week 2 2022>

| 1 | (Child* or pediatric* or paediatric* or teen* or adolesc* or youth* or young adult* or young people or young person).ti,ab. | 984037 |
| --- | --- | --- |
| 2 | (advisory board* or advisory committee* or advisory group* or SPOR or strategy for patient-oriented research or patient-centred outcomes research institute or co-design or youth-adult partnership* or community based participatory research).ti,ab. | 4306 |
| 3 | ((Patient* or famil* or parent* or caregiver* or stakeholder* or community or youth* or child* or adolesc* or public or consumer) adj2 (involv* or oriented or orientated or collaborat* or partner* or collaborat* or engage* or activat*) adj4 (research or trial or method* or health technology assessment or study)).ti,ab. | 7867 |
| 4 | action research/ | 3527 |
| 5 | patient participation/ | 2736 |
| 6 | ((mental or psychiatric or psychologic*) adj (health or illness* or disorder* or disease* or problem or problems or issue* or concern* or well-being)).ti,ab. | 342584 |
| 7 | mental health services/ | 36969 |
| 8 | mental health/ | 77629 |
| 9 | anxiety disorders/ or acute stress disorder/ or castration anxiety/ or death anxiety/ or generalized anxiety disorder/ or obsessive compulsive disorder/ or panic disorder/ or posttraumatic stress disorder/ or separation anxiety/ or phobias/ or acrophobia/ or agoraphobia/ or claustrophobia/ or ophidiophobia/ or school phobia/ or social phobia/ or anxiety/ or computer anxiety/ or mathematics anxiety/ or performance anxiety/ or social anxiety/ or speech anxiety/ or test anxiety/ or panic attack/ or panic/ or panic disorder/ or compulsions/ or repetition compulsion/ or obsessions/ or obsessive compulsive personality disorder/ or neurosis/ or childhood neurosis/ or experimental neurosis/ or occupational neurosis/ or traumatic neurosis/ or adjustment disorders/ or coping behavior/ or adjustment/ or exp emotional adjustment/ or occupational adjustment/ or school adjustment/ or social adjustment/ | 269151 |
| 10 | affective disorders/ or affective psychosis/ or bipolar disorder/ or cyclothymic personality/ or major depression/ or anaclitic depression/ or dysthymic disorder/ or endogenous depression/ or postpartum depression/ or reactive depression/ or recurrent depression/ or treatment resistant depression/ or atypical depression/ or "depression (emotion)"/ or seasonal affective disorder/ | 204740 |
| 11 | stress/ or chronic stress/ or environmental stress/ or occupational stress/ or psychological stress/ or social stress/ or stress reactions/ or emotional trauma/ | 128739 |
| 12 | somatoform disorders/ or body dysmorphic disorder/ or hypochondriasis/ or neurasthenia/ or neurodermatitis/ or somatization disorder/ or somatoform pain disorder/ or conversion disorder/ or hysterical paralysis/ or hysterical vision disturbances/ or pseudocyesis/ or somatization/ or hysterical paralysis/ or histrionic personality disorder/ or malingering/ or factitious disorders/ or trichotillomania/ | 19450 |
| 13 | psychosis/ or acute psychosis/ or affective psychosis/ or childhood psychosis/ or chronic psychosis/ or experimental psychosis/ or hallucinosis/ or "paranoia (psychosis)"/ or reactive psychosis/ or schizophrenia/ or paranoid schizophrenia/ | 118771 |
| 14 | eating disorders/ or anorexia nervosa/ or bulimia/ or hyperphagia/ or "purging (eating disorders)"/ or binge eating/ | 32818 |
| 15 | self destructive behavior/ or attempted suicide/ or head banging/ or self inflicted wounds/ or self injurious behavior/ or self mutilation/ or suicide/ or suicide prevention/ or suicidal ideation/ | 51191 |
| 16 | (eating disorder* or anorexia nervosa or bulimi* or binge eat* or (self adj (injur* or mutilat*)) or suicide* or suicidal or parasuicid* or mood disorder* or affective disorder* or bipolar i or bipolar ii or (bipolar and (affective or disorder*)) or mania or manic or cyclothymic* or depression or depressive or dysthymi* or neurotic or neurosis or adjustment disorder* or antidepress* or anxiety disorder* or agoraphobia or obsess* or compulsi* or panic or phobi* or ptsd or posttrauma* or post trauma* or combat or somatoform or somati#ation or medical* unexplained or body dysmorphi* or conversion disorder or hypochondria* or neurastheni* or trichotillomania or affective symptoms or mental disorder* or mental health or psychosis or psychotic).ti. | 368348 |
| 17 | 2 or 3 or 4 or 5 | 17437 |
| 18 | 6 or 7 or 8 or 9 or 10 or 11 or 12 or 13 or 14 or 15 or 16 | 975769 |
| 19 | 1 and 17 and 18 | 1041 |
| 20 | limit 19 to yr="2000 -Current" | 944 |
